# Web-Based Survey Piloting Process as a Model for Developing and Testing Past Contraceptive Use and Pregnancy History: Cystic Fibrosis Case Study

**DOI:** 10.2196/46395

**Published:** 2023-07-18

**Authors:** Emily M Godfrey, Malaika R Schwartz, Karen D Hinckley Stukovsky, Danielle Woodward, Amalia S Magaret, Moira L Aitken

**Affiliations:** 1 Departments of Family Medicine and Obstetrics and Gynecology University of Washington Seattle, WA United States; 2 Department of Family Medicine University of Washington Seattle, WA United States; 3 Collaborative Health Studies Coordinating Center Department of Biostatistics University of Washington Seattle, WA United States; 4 Survey Research Division Social Development Research Group University of Washington Seattle, WA United States; 5 Seattle Children's Research Institute Division of Pulmonary and Sleep Medicine, Department of Pediatrics University of Washington Seattle, WA United States; 6 Division of Pulmonary, Critical Care and Sleep Medicine Department of Medicine University of Washington Seattle, WA United States

**Keywords:** contraception, chronic disease, genetic disease, questionnaire reliability, surveys and questionnaires, contraceptive, birth control, cystic fibrosis, reproductive health, pregnancy, electronic survey, obstetrical history

## Abstract

**Background:**

Individuals with complex, chronic diseases are now living longer, making reproductive health an important topic to address in the health care setting. Self-respondent surveys are a feasible way to collect past contraceptive use and pregnancy history to assess contraceptive safety and effectiveness. Because sensitive topics, such as contraception and pregnancy outcomes, can vary across social groups or cultures, piloting questions and evaluating survey administration procedures in the target population are necessary for precise and reliable responses before wide distribution.

**Objective:**

This study aimed to develop a precise and reliable survey instrument and related procedures among individuals with cystic fibrosis regarding contraceptive use and obstetrical history.

**Methods:**

We piloted and tested web-based questions related to contraceptive use and pregnancy history among 50 participants with and those without cystic fibrosis aged 18 to 45 years using a 3-tier process. Findings from each tier informed changes to the questionnaire before testing in the subsequent tier. Tier 1 used cognitive pretesting to assess question understanding and the need for memory prompts. In tier 2, we used test-retest self- and interviewer-administered approaches to assess question reliability, evaluate response missingness, and determine confidence between 2 types of survey administration approaches. In tier 3, we tested the questionnaire for clarity, time to complete, and whether additional prompts were necessary.

**Results:**

In tier 1, respondents suggested improvements to the web-based survey questions and to the written and visual prompts for better recall regarding past contraceptive use. In tier 2, the test-retest reliability between self- and interviewer-administrative procedures of “ever use” contraceptive method questions was similar, with percent absolute agreement ranging between 84% and 100%. When the survey was self-administered, the percentage of missing responses was higher and respondent confidence about month and year when contraceptive methods were used was lower. Most respondents reported that they preferred the self-administered survey because it was more convenient and faster to complete.

**Conclusions:**

Our 3-tier process to pilot web-based survey questions related to contraceptive and obstetrical history in our complex disease population helped us tailor content and format questions before wide dissemination to our target population. Results from this pilot study informed the subsequent larger study design to include a 10% respondent test-retest self- and interviewer-administered quality control component to better inform imputation procedures of missing data.

## Introduction

Individuals with complex, chronic diseases, like cystic fibrosis (CF), which is a genetic, multiorgan disease, are increasingly living into adulthood because of modern medical advances [1]. The number of adult women with CF in the United States has nearly doubled over the last 3 decades from 4500 to approximately 8000 [1]. Although evidence suggests that adults with CF have similar rates and ages of initiation of sexual intercourse as the general population, reproductive health, including contraceptive care, remains an overlooked health care need [2-4]. Unintended pregnancy in this population is common, with as many as 50% reporting at least 1 unplanned pregnancy in their lifetime [5]. As with many complex, chronic diseases, unintended pregnancy is undesirable in CF. Research suggests that postpartum individuals with CF have worsening disease conditions due to weight loss and pulmonary decline compared with prepregnancy levels [6,7]. Contraceptive use remains one of the key ways to prevent unintended pregnancies. However, data are limited regarding its safety and efficacy in persons living with CF [8].

The US Medical Eligibility for Contraceptive Use (MEC), a national contraceptive guideline for health care providers, includes over 1800 recommendations for more than 60 conditions and characteristics, as well as information on certain drug interactions [9]. The US MEC provides recommendations for CF, but the current body of evidence is limited due to design flaws, few outcomes, and small sample sizes [8,10]. Thus, the US MEC states that the guidelines for CF are appropriate only for persons with CF for whom “no other conditions are present,” which limits their recommendations’ generalizability to healthier people with CF [8]. Potential complications for patients with CF include decreased pulmonary function, CF-related diabetes, CF-associated liver disease, reduced renal function, increased risk for hypertension, and poor bone health [11-14]. There are theoretical concerns that hormonal contraception may adversely impact these complications.

A nationwide observational trial entitled Contraceptive Assessment of Safety and Effectiveness in CF (CASE.4.CF) for patients with CF was initiated through the University of Washington in 2020. This population-based trial seeks to determine contraceptive safety and efficacy related to pulmonary function, diabetes, bone health, liver and gallbladder disease, and concomitant medication use in CF by linking past contraceptive exposure and obstetrical history with clinical outcomes. Episodic and annual health outcomes for individuals with CF are tracked closely by the Cystic Fibrosis Foundation Patient Registry (CFFPR), a robust registry that covers approximately 95% of people in the United States living with CF [15]. Unfortunately, contraception is not captured in the CFFPR. Only 1 question is asked in the registry about whether a pregnancy occurred in the last year, while pregnancy outcomes are not included.

Our research team considered potential sources to obtain contraceptive exposure data, such as regional and national practice-based or patient-centered networks, which allow data from different electronic medical record platforms to be extracted en masse through a single data sharing system. However, without a linked pharmacy database to verify contraceptive prescriptions, misclassification of contraceptive exposure was likely [16], and neither source included over-the-counter methods such as condoms or natural family planning. Most population-based data that link prior use of contraception with health outcomes in the United States use self-respondent surveys [17-21]. These survey questions serve as generic prototypes for how to ask about past contraceptive use in the general population. However, willingness to provide responses to sensitive topics, such as contraceptive and pregnancy history, sexual health, and substance use, can vary across social groups or cultures [22,23]. In this pilot study, we sought to create and test survey items and procedures related to past contraceptive use and pregnancy history for completeness and reliability of responses using a 3-tier piloting process.

## Methods

### Study Overview

We conducted this study to pilot questions before wide dissemination in the CASE.4.CF study of more than 500 participants in our target population. This pilot study was conducted at the University of Washington between February 2020 and June 2020 and included administration of a survey with web-based video or phone interviews among 50 people in 3 separate tiers (Table 1). Findings from each tier informed changes to the subsequent tier before full-scale administration.

**Table 1:** Outline of three study tiers (N=50).

Tier	Respondents, n	Purpose	Goal
1	10	Cognitive pretesting	Check question wording and meaning Assess if responses are adequate
2	19	Response reliability	Assess whether self-report or interviewer-administered surveys of past contraceptive use and medical and pregnancy history provides consistent answers when surveys taken 2 weeks apart Determine whether self-respondent web-based or interviewer-administered surveys promote more accurate responses
3	21	Pilot survey completion timing	Determine time it takes to complete the total survey Assess whether any remaining questions are unclear Assess adequacy of choice of responses

### Survey Instrument

We designed the questionnaire with input from CASE.4.CF investigators. We based our draft questions on nationally representative survey instruments that asked about prior contraceptive use and pregnancy history, including the Nurses’ Health Study, National Survey of Family Growth, Pregnancy Risk Assessment Monitoring System, Behavioral Risk Factor Surveillance System, and the Women Cares Study [17,18,20,21,24]. To assess the long-term safety of contraceptive use in our population, we sought to obtain information about contraceptive exposure going as far back as 2008 up to 2020. Questions related to contraception started by asking about “lifetime” use for each method, followed by a breakout of specific time intervals (month and year of start and stop dates) to determine total duration and amount of overlap between methods. We also asked about the use of hormonal contraception for reasons other than pregnancy prevention because indication questions have been shown to have better responses than open-ended questions [25]. We included techniques that enhance recall and completeness of data, including (1) listing specific contraceptive methods, (2) introducing memory aids (also known as showcards) with pictures and brand names of different contraceptive methods, and (3) providing definitions of pregnancy outcomes (eg, miscarriage as being pregnancy loss before 21 weeks and stillbirth as pregnancy loss after 21 weeks). Contraceptive methods surveyed included estrogen-containing and progestin-only birth control pills; contraceptive patch; vaginal ring; 3-month injectable, intrauterine devices; subdermal implants; and nonhormonal methods, including condoms, cervical caps, spermicide, withdrawal, lactational amenorrhea, and fertility awareness methods. Pregnancy history questions included whether the respondent had ever been pregnant, how many times, the outcome of each pregnancy, and whether the pregnancy was planned or unplanned. The survey also included questions about menstrual and past medical and surgical histories to assess if a respondent was not at risk for pregnancy or not a candidate for hormonal contraceptive use.

### Data Collection Modality

The specific method by which a survey is administered to respondents is a potential source of measurement error, particularly for sensitive topics, such as past contraceptive use, in which the presence of an interviewer may be undesirable and awkward for respondents [26]. Self-administered surveys offer a potentially higher response rate but can also contribute to response error because they assume that respondents understand each question as intended and can independently navigate the survey tool alone [23]. Therefore, we drafted 2 separate questionnaires based on the data collection modality: one self-administered and the other interviewer-administered. We included the interviewer-administered approach because most nationally representative contraceptive surveys like National Survey of Family Growth use an interviewer [27]. We anticipated that the detailed recollection of contraceptive use going as far back as 12 years would be more thoroughly obtained through interviewer prompting. Additionally, including an interviewer would allow for the use of more detailed, dynamic prompts to build a narrative about contraceptive use rather than static, web-based survey questions. On the other hand, because CF is a rare disease and maintaining anonymity of patients is more challenging, we were concerned about the potential for social desirability bias with an interviewer present [26,28]. As such, we considered a combination of administrative procedures, with some questions being interviewer-administered and others completed independently to increase response rates, while maintaining response accuracy.

### Sampling Frame and Recruitment

Following the precedents of piloting and validating studies conducted with between 20 and 50 people [26], we enrolled 50 English-speaking women with or without CF, aged 18 to 45 years. We included several women without CF because of our desire to avoid premature familiarity of the survey questions within our targeted population for the larger survey study. Because individuals with CF have a lifelong disease, take multiple medications daily, and regularly see their health care professionals several times a year, many within this population are known to maintain years’ worth of medical visit notes and medication lists. Thus, to diversify our sample to include those who may not keep detailed notes and thus not remember as much about their contraceptive use going as far back as 2008, we included a few respondents who did not have CF [23,29,30]. We assumed that individuals without CF would not have kept detailed notes of their medical history going as far back as 2008.

We recruited potential participants from several CF organizations, including Cystic Fibrosis Research Inc, Cystic Fibrosis Reproductive and Sexual Health Collaborative, and the Cystic Fibrosis Foundation Community Voice program. Each of these organizations reached individuals who had voluntarily registered with their organization to be contacted for potential research opportunities, were of reproductive age, and were US residents. The CF organizations helped recruit participants by sending an email introducing the study and a link to an eligibility survey for interested members. Potential participants completed the eligibility survey with questions about whether they had CF, their assigned sex at birth, age, prior pregnancies, and whether contraception had been used in their lifetime. They were contacted via email by the research study staff if eligible.

### Study Procedures

We pretested and piloted our draft web-based survey in 3 separate tiers with 50 unique participants (Table 1). Participants were permitted to participate in 1 tier only and were paid US $30 for their time. After completing the study procedures for each tier, the research team analyzed the findings and revised the survey accordingly before initiating the procedures outlined in the next tier. Once tier 1 was complete, we programmed the survey for tiers 2 and 3 as a web-based questionnaire using REDCap (Research Electronic Data Capture), a secure web-based application designed to support data capture for research studies [31].

We tested our initial survey questions using several different pretest survey methods (Textbox 1) [23]. For tier 1, we chose cognitive interviewing as our approach to pretest the contraception questions and determine if a respondent interpreted each question as intended. Research staff contacted eligible participants by email to set up a time for the interview and conducted the interview either over the phone or via Zoom, depending on each study participant’s preference.

In tier 2, we conducted test-retest reliability by asking each participant to complete both a self- and an interviewer-administered survey 2 weeks apart, with half the group starting with the self-administered survey and the other half starting with the interviewer-administered survey. We chose 2 weeks to reduce recall bias because we were unable to compare participant responses against a “gold standard,” like a pharmacy or medical record database [25]. After each interview, we also asked participants to give feedback on (1) content: ease, comfort, content clarity, ability to recall, and confidence in the accuracy of their responses; (2) preferences regarding survey administration; and (3) overall length of the questionnaire. Participants who completed the self-administered survey first received a link via email by the research staff and were given 1 week to complete the survey before the staff contacted them to set up a time to participate in the interviewer-administered survey by Zoom or phone 2 weeks after completing the web-based survey. For participants who completed the interviewer-administered survey first, similar processes were followed but in reverse.

In tier 3, we assessed time to complete the entire web-based survey and gathered written feedback about questions they perceived as unclear. Each participant was sent a link to the self-administered web-based survey via email.

**Piloting and validating study procedures of each section of questionnaire, by tier.**

**Tier 1**

- Participants were provided a digital showcard (pictures) and the proposed study survey questions with instructional prompts and response options before and during the interview
- We asked each respondent to “think out loud” about the showcards, questions, and responses, but not necessarily to answer the question. A note taker took field notes during the interview process. We focused on the following:
  - Respondent comprehension: Does the showcard and question make sense? Does respondent understand the question or what the showcard is depicting? How is respondent interpreting the question? Are there definitions needed to answer the question?
  - Difficulty of task: How easy or difficult is it to answer the question? What additional transitions, examples, memory prompts, or introductions are needed?

**Tier 2**

- To measure consistency over time, we had the respondents who completed the same survey twice, 2 weeks apart:
  Half of the participants answered a self-administered (SELF) web-based survey first then participated in an interview
  The other half of the participants took part in an interview first and then completed a SELF web-based survey
- For the SELF web-based survey, respondents were emailed consent information and the show cards, along with the survey link. They were given 1 week to complete the survey, with a reminder sent 3 days after the initial email was sent. Once the web-based questionnaire was complete, the research team contacted them to schedule an interview 2 weeks later either over the phone or via Zoom
- For the interviewer-administered (IA) survey, the interviewer reviewed the information sheet, confirmed participation, and obtained verbal consent to audio-record the interview. After getting consent, the interviewer administered the survey, using showcards for the respondent to reference throughout the interview. A note taker took notes on requests for clarification, difficulty answering a question, requests to reread the questions, and questions that required a probe. The note taker assessed for:
  - Accuracy: Is respondent able to accurately recall the information and report what we want?
  - Useful content: Is the information we are getting useful? Is this the data that we want?
  - Ease of administration: Is the respondent understanding the question when the question is read to them? Is the data collection instrument easy to administer? Are the responses complete?
- For content validity, each respondent was asked a few debriefing questions at the end of the SELF or IA survey regarding the process. They were asked about:
  - How did you like or dislike the survey modality?
  - Was the questionnaire comprehensive (was contraceptive topic adequately covered)?
  - Were the questions clear and easy?
  - Were there questions that you feel may have violated your privacy?
  - Was the questionnaire too short, long, or about right?
  - How confident were you when you responded to the start and stop dates of your birth control method use?

**Tier 3**

- For the web-based SELF survey, respondents were asked to do the following:
  - Provide answers to the questions (time to complete was captured via the web)
  - Give feedback at the end on 5 key questions, including showcard depictions, helpful prompts for remote recall of start and stop dates of each birth control method, whether any questions were unclear, and if response options were adequate

### Analysis

#### Analysis—Tier 1

We reviewed the comments from the note taker for each cognitive interview and collated them. To identify potential survey improvements, 2 researchers independently coded the responses into themes, such as “pictures helpful” or “would not remember.” The researchers compared coding themes, discussed and reconciled differences. The senior researcher helped resolve coding discrepancies until the team reached consensus. We adjusted a survey question when 50% or more of the coded responses had a prevailing theme.

#### Analysis—Tier 2

In tier 2 where participants took the survey 2 times and 2 weeks apart to assess the reliability of birth control information given, we tallied the number of discordant responses between self- and interviewer-administered survey responses and calculated the percent agreement for each question. We also calculated κ statistic to measure the accuracy of participant reporting of birth control history [32]. We considered κ results between 0.81 and 1.00 as perfect agreement, 0.61 and 0.80 as substantial agreement, 0.21 and 0.60 as fair agreement, and 0.01 and 0.20 as poor agreement [33].

#### Analysis—Tier 3

The final draft was evaluated in terms of completion time using the start and end times automatically recorded by REDCap. We removed outliers, as they could have been the result of leaving a browser open and not completing the survey until later. Similar to our process during tier 1, we coded the open-ended feedback responses. Themes that met a minimum 50% of responses were used to guide final changes to the survey questions.

### Ethics Approval

This study was approved by the University of Washington Human Subjects Division as an exempt study (ID: STUDY00009033). All participants were provided a web-based informed consent form and agreed to participation before study enrollment.

## Results

In each tier, the majority of respondents had CF, with <15% of participants without CF. We enrolled 10 participants into our pretesting tier (tier 1). We describe changes made in the instrument after this round of input (Textbox 2). For example, each participant preferred the term “birth control” to “contraception.” Respondents made other suggestions about birth control questions specifically: ask about use starting in 2008 going forward in time (vs starting with present day first, going back in time), allow participants to select birth control options by type (eg, pills, implants, and barrier methods), and provide more explicit images of certain methods (eg, withdrawal method). Most participants preferred prompts to help them remember when they used different methods of birth control, including recalling life events such as when they were in college, married, or had just given birth.

In tier 2, a total of 19 participants completed both the self- and interviewer-administered surveys 2 weeks apart. Together, the 19 participants used 15 separate types of birth control. Each participant on average used 4 separate methods. The percent absolute agreement ranged from 84% to 100% for “ever use,” depending on method type. The 2 most commonly “ever” used birth control methods included condoms (n=17) and combined birth control pills (n=16). Among the 19 respondents, 17 were concordant with “ever” condom use (89% agreement; κ=0.60, 95% CI 0.15-1.00), with 2 respondents equally discordant about ever use on the self- and interviewer-respondent surveys. Sixteen respondents were concordant with “ever use” combined hormonal pills (84% agreement; κ=0.63, 95% CI 0.21-1.00), but 3 respondents who did not report having used pills on the self-administered survey did so on the interviewer-administered survey (Table 2).

For both condoms and birth control pills, the percentages of responses missing when asked about specific start and stop dates were greater for those completing the survey alone than when completing the survey with an interviewer (Table 3).

Respondents reported feeling more confident about their start and stop date responses with the interviewer-administered survey compared with the self-administered survey (overall 58% agreement; κ=0.28, 95% CI −0.05 to 0.61; Table 4). Three respondents who reported being very or somewhat uncertain on the self-administered survey were either somewhat or very certain about their responses on the interviewer-administered survey. Four individuals, who reported being somewhat certain about their responses on the self-administered survey, were very certain about their responses on the interviewer-administered survey. Only 1 individual had a relative decrease in certainty when completing the survey with an interviewer.

**Tier 1—feedback summary (N=10).**

**Birth control section**

- Prefer the term “birth control” over “contraception”
- Provide birth control pictures and brand names to jog memory
- Many may be less familiar with some methods like withdrawal, rhythm method, and breastfeeding (lactational amenorrhea). Will need to define
- Provide prompts to remember birth control start and stop date
- Tell respondents to have their medical records available when doing this survey
- Provide birth control summary at the end to review and ensure accuracy

**Pregnancy history section**

- “Miscarriage” and “stillbirth” are considered emotional terms. Frame questions with sensitivity
- “Abortion” was understood by most respondents, but suggested alternative terms like “termination of pregnancy”
- All respondents said they would not have difficulty remembering the outcomes of their pregnancies
- Provide definitions of “planned” vs “unplanned” (pregnancy)
- Ask permission before summarizing pregnancy outcomes. Concern that those who have had miscarriages or stillbirths may be overwhelmed
- Have counselors available for respondents who find survey questions upsetting

**Table 2:** Tier 2—proportions of assignment, Cohen κ, and percent absolute agreement of interviewer-administered (IA) and self-administered (SELF) among 19 female respondents with CF (“ever-use”).

Contraceptive method	Proportionate distribution of assignment^a^					Cohen κ^b^ (95% CI)		Percent absolute agreement^c^ (%)
	IA Yes, SELF Yes	IA No, SELF No	IA Yes, SELF No	IA No, SELF Yes				
Birth control pills (estrogen and progestin)	12	4	3	0	0.63 (0.21-1)		84	
Contraceptive patch	2	17	0	0	1.00 (1-1)		100	
Vaginal ring	—^d^	—	—	—	—		—	
Birth control pills (progesterone only)	2	17	0	0	1.00 (1-1)		100	
Copper IUD^e^ (nonhormonal IUD)	3	16	0	0	1.00 (1-1)		100	
Hormonal IUD	4	15	0	0	1.00 (1-1)		100	
Arm implant	1	17	1	0	0.64 (0.22-1)		95	
Depo-Provera shot	2	17	0	0	1.00 (1-1)		100	
Condoms (male or female condoms)	15	2	1	1	0.60 (0.15-1)		89	
Diaphragm or cervical cap	—	—	—	—	—		—	
Spermicide (includes gel, foam, suppository, or Today Sponge)	1	18	0	0	1.00 (1-1)		100	
Withdrawal or pulling out	11	7	0	1	0.89 (0.44-1)		95	
Fertility awareness method (period-tracker app, rhythm method, and natural family planning)	6	12	1	0	0.88 (0.44-1)		95	
Breastfeeding (lactational amenorrhea)	3	13	0	3	0.58 (0.17-0.99)		84	
Abstinence	5	12	0	2	0.76 (0.32-1)		89	
Emergency contraception pills	5	14	0	0	1.00 (1-1)		100	

^a^Proportions may not add to 1 due to rounding.

^b^Ranges from −1 to 1.

^c^Ranges from 0% to 100%.

^d^Unable to calculate—no users reported having used these methods.

^e^IUD: intrauterine device.

**Table 3:** Tier 2—proportions of responses missing when asked about contraceptive start and stop month or year of interviewer-administered (IA) and self-administered (SELF) among 19 female respondents with cystic fibrosis (only 2 most commonly used birth control methods shown).

Contraceptive		Year of use	Month of use
**Condoms (n=17)^a^**			
	SELF	23.9% (11/46)	63.3% (19/30)
	IA	22.2% (10/45)	16.7% (5/30)
**Birth control pills (n=16)^a^**			
	SELF	39.2% (20/51)	53.6% (15/28)
	IA	2.0% (1/51)	3.6% (1/28)

^a^Raw numbers do not add up because some women used methods multiple times over a period of 12 years.

**Table 4:** Tier 2—proportions of assignment regarding self-reported confidence in response about contraceptive stop and start dates, Cohen κ, and percent absolute agreement of interviewer-administered and self-administered among 19 female respondents with cystic fibrosis.^a^

Self-administered	Interviewer-administered					Total
	Very uncertain	Somewhat uncertain	Somewhat certain	Very certain		
Very uncertain	0	0	1	1	2	
Somewhat uncertain	0	0	0	1	1	
Somewhat certain	0	0	7	4	11	
Very certain	0	0	1	4	5	
Total	0	0	9	10	19	

^a^Observed agreement: 58%, =0.28, 95% CI −0.05 to 0.61.

Despite feeling more certain about their responses when responding to an interviewer-administered survey, the majority of respondents indicated they preferred taking the survey by themselves. We found that self-administered surveys took an average of 11 minutes to complete, whereas the interviewer-administered surveys took an average of 16 minutes. Participants gave more detailed answers about their stop and start dates, providing month and year (not just year) in the interviewer-administered survey.

In tier 3 (n=21), all of the respondents indicated that the questions in each section were clearly written. Several participants were uncertain how to answer birth control start and stop date questions for condoms, abstinence, and withdrawal, as those methods are often used intermittently. When asked if the showcard pictures and descriptions of methods of surgery and birth control were useful, 50% (11/21) and 62% (13/21) respectively affirmed usefulness. Participants said that the birth control showcard helped them differentiate between different birth control pills, helped jog their memory, or remember what brand they used. The final survey questions related to past contraceptive use and pregnancy history are listed in Multimedia Appendix 1.

## Discussion

### Principal Results

Our 3-tier piloting and testing process found that question wording, how we ordered questions, and clarity of depictions helped increase precision and completeness of our contraceptive health survey for this complex disease population. We found that the majority of respondents considered pictures of contraceptive methods helpful. Although participants reported a preference for answering the questions by themselves, respondents reported more complete birth control history responses, and with greater confidence, when an interviewer administered the survey. Our findings from this pilot study helped inform the study design for the subsequent larger CASE.4.CF study in our target population to include a 10% respondent test-retest self- and interviewer-administered quality control component to better inform imputation procedures of missing data. Responses from this larger study will be used to calculate contraceptive exposure and linked to select clinical outcomes in the CFFPR once data collection is complete.

### Comparison With Prior Work

Precise ascertainment of past contraceptive use to assess contraceptive exposure is crucial to determine whether these methods are safe in individuals with complex medical conditions. The most ideal study design is a prospective one that follows people longitudinally. However, the utility of this approach is questionable, especially if adverse events are rare. Most studies directing the recommendations in national guidelines are generated from evidence provided by retrospective cohort and case-control study designs, which are provided by large population-based administrative health care databases [34]. In the United States, administrative databases that contain information about specific health outcomes related to rare medical conditions are mostly limited to disease-specific registries in which contraceptive use is not collected.

Most population-based surveys collecting past contraceptive use in the United States have used an interviewer [20,21]. Self-administered surveys are not considered ideal because of potential measurement errors, which can be minimized with pretested and piloted questions [23]. For example, patients who do not remember past contraceptive use will be misclassified unless their responses can be verified. Medication exposure recall is negatively influenced by the number of daily medications, which is applicable to patients with CF who on average take 7 medications each day [25,35,36]. Because of the uniqueness of this population living with time-consuming daily medication regimens [37] and the uncertainty of biases that may surround sensitive questions related to sexual and reproductive health, we included a component to evaluate the survey administration approach as one of the tiers in our pilot testing.

To our knowledge, this is one of the first studies to evaluate a stepwise process to pilot contraception and pregnancy survey questions among a rare disease population. Most survey research literature stresses the importance of piloting questions before wide dissemination [23,38]. Untested questions that are poorly worded can lead to inaccurate information about important medical questions that health care providers need to practice evidence-based medicine. Yet, despite the importance of piloting survey questions before wide dissemination, we found little guidance in the literature regarding how best to approach pretesting and piloting survey questions related to sexual and reproductive health in the patient population. One study describing the development and piloting of contraceptive questions for pharmacists in Canada used theories grounded in implementation science [39]. Because the primary aim of CASE.4.CF is to determine contraceptive exposure over time, methods used to pilot questions in the Canadian study were not applicable to our patient population.

### Limitations

This study has limitations. As a rare disease, we were reluctant to pilot our survey entirely among individuals with CF out of concern that this might limit the number of participants in our targeted population for the larger study once the survey was finalized and disseminated. Because our recruitment was primarily through CF organizations, the majority of participants who enrolled in this pilot phase had CF. Additionally, we did not compare survey responses to medical or pharmacy records, which would provide some verification of prescribed methods. The historical time period in question may have impacted the accuracy of results, as it is difficult for participants to recall questions about contraceptive use in the last 12 years. Shorter recall periods are desirable for the increased accuracy of contraceptive use, particularly with methods that are used on and off again, such as condoms.

### Conclusions

Our 3-tier stepwise process to pilot a web-based contraceptive and obstetrical survey questions helped us tailor content and format for optimal data capture before wide dissemination of a contraceptive and obstetrical survey in the CF population, which is currently underway. This study adds to the literature with a description of the process we used to develop and pilot a contraceptive and obstetrical survey instrument among individuals with CF. This process can serve as a model for survey development and piloting for other complex, chronic disease populations.

## Appendix

Multimedia Appendix 1 Final survey questions used for CASE.4.CF Study.

## Abbreviations

CASE.4.CF: Contraceptive Assessment of Safety and Effectiveness in CF
CF: cystic fibrosis
CFFPR: Cystic Fibrosis Foundation Patient Registry
MEC: Medical Eligibility for Contraceptive Use
REDCap: Research Electronic Data Capture

## Data Availability

The data sets generated or analyzed during this study are available from the corresponding author upon reasonable request.
