# Supplementary material for: Web-Based Survey Piloting Process as a Model for Developing and Testing Past Contraceptive Use and Pregnancy History: Cystic Fibrosis Case Study
Source: JMIR Form Res. 2023 Jul 18;7:e46395. doi: 10.2196/46395 (PMC10394597; doi:10.2196/46395)
Supplement: Multimedia Appendix 1 [file formative_v7i1e46395_app1.pdf]

Introduction

Thank you for agreeing to participate in this study. Your responses will help us provide CF providers with information on the health impacts of contraceptives on women with CF.

Medical History

The first questions are about your medical history regarding blood clots and smoking

Blood Clot Questions

Have you ever used blood thinners for any reason? This may include heparin, aspirin, or other types of blood thinners.	Select one:<br>*1. Yes*<br>*2. No*
If (1) selected<br>*Please give the reason for using blood thinners*	
Have you ever been diagnosed with having a blood clot that required taking blood-thinning medications (e.g., Aspirin, Coumadin or Lovenox)?	Select one:<br>*1. Yes*<br>*2. No* [continue to next section]
If (1) selected<br>*How many times have you been diagnosed with a blood clot that required you to take a blood thinner?*	Select one:<br>*1*<br>*2*<br>*3*<br>*4*<br>*5 or more*
1st blood clot: Do you know the year you were diagnosed?	Select one:<br>*1. Yes* [enter year]<br>*2. No, but I know how old I was at the time* [enter age]<br>*3. No*
If (1) selected<br>*1st blood clot: Was the clot though to be associated with:*	Select one:<br>*1. Central line (PORT)*<br>*2. Peripherally inserted central catheter (PICC)*<br>*3. Trauma-related*<br>*4. Underlying genetic blood clot disorder*<br>*5. Unsure/Don't know*<br>*6. Other*
If (6) selected<br>*Specify 1st blood clot reason*	
Repeat questions for next blood clot, up to 5 blood clots	

Smoking Questions

Have you ever smoked any type of tobacco? This includes cigars, cigarettes, vapes (e-cigarettes), and pipes.	Select one:<br>*1. Yes*<br>*2. No* [continue to next section]
If (1) selected	Select one:

Do you currently smoke any type of tobacco? This includes cigars, cigarettes, vapes (e-cigarettes), and pipes.	*1. Yes*<br>*2. No*

Medication History

Next, we have some questions about any cystic fibrosis medications you may have taken

Are you currently using any CFTR modulators (e.g., Kalydeco, Orkambi, Symdeko, or Trikafta), as prescribed by your doctor or as part of a study?	Select one:<br>*1. Yes*<br>*2. No*
If (2) selected:<br>*Have you taken a CFTR modulator in the past?*	Select one:<br>*1. Yes*<br>*2. No* [continue to next section]
If (1) selected for either of the previous questions<br>*Which modulator?*	Select one:<br>*1. Kalydeco = Ivacaftor (VX770) Approved 01/2012*<br>*2. Orkambi = Ivacaftor (VX770)+ Lumacaftor (VX 809) Approved 07/2015*<br>*3. Symdeko = Ivacaftor (VX770)+ Tezacaftor (VX661) Approved 02/2018*<br>*4. Trikafta = Ivacaftor (VX770)+ Tezacaftor (VX661)+Elexacaftor (VX445) Approved 10/2019*<br>*5. Can't remember which modulator*
Date you started taking the modulator?	Select one:<br>*1. I know the year and month* [enter year and month]<br>*2. I know the year* [enter year]<br>*3. I know how old I was* [enter age]<br>*4. I don't know*
Date you stopped taking the modulator?	Select one:<br>*1. I know the year and month* [enter year and month]<br>*2. I know the year* [enter year]<br>*3. I know how old I was* [enter age]<br>*4. I don't know*
Did you take a modulator before this one?	Select one:<br>*1. Yes* [repeat questions for next modulator, up to 5 modulators]<br>*2. No*

Surgical History

The next questions are about your surgical history

Have you ever had some kind of surgery that would impact your ability to get pregnant (e.g., tubal ligation, sterilization, hysterectomy, or ovary removal?	Select one:<br>*1. Yes*<br>*2. No* [continue to next section]

[includes description and image for each procedure]	
If (1) selected<br>*What surgery have you had that would impact your ability to get pregnant?*	Select all that apply:<br>*1. Tubal ligation (both of your tubes tied, cut, or removed)*<br>*2. Tubal sterilization procedure called "Essure"*<br>*3. Hysterectomy (surgery to remove your uterus)*<br>*4. Oophorectomy (both your ovaries removed)*<br>*5. Other*<br>*6. Prefer not to answer*
If (5) selected<br>*Other (Please specify)*	
When did you have the surgery?<br>*(If more than one surgery, give earliest date)*	Select one:<br>*1. I know the year and month*<br>*2. I know the year*<br>*3. I know how old I was*<br>*4. I don't know*
If (1) or (2) selected<br>*What year did you have the surgery?*<br>*(If more than one surgery, give earliest date)*	
If (1) selected<br>*What month did you have the surgery?*<br>*(If more than one surgery, give earliest date)*	
If (3) selected<br>*How old were you when you had the surgery?*<br>*(If more than one surgery, give youngest age)*	

Menstruation

In this next section, we are going to ask you some questions about your menstrual history.

How old were you when you first got your period?	
Do you (still) get your regular, monthly period?	Select one:<br>*1. Yes* [continue to next section]<br>*2. No*
If (2) selected<br>*What do you think is the main reason that you are not having a period?*	Select one:<br>*1. You are transitioning to menopause (perimenopause)*<br>*2. You have reached menopause (you have not had a period in over a year AND were not on birth control)*

	3. You have had a hysterectomy<br>*4. You are currently, or were recently, pregnant*<br>*5. You are currently breastfeeding*<br>*6. You are currently using a form of birth control that limits or changes menstruation*<br>*7. You are underweight*<br>*8. You are taking Megace*<br>*9. Unsure/Don't know*<br>*10. Other*
If (10) selected<br>*Other (please specify)*	
If (2) selected<br>*How old were you when you stopped having periods for more than a year (and were not on hormonal birth control)?*	Select one:<br>*1. I know how old I was at the time* [enter age]<br>*2. I know the year and month* [enter year and month]<br>*3. I know the year* [enter year]

Birth Control History

Now we want to ask you about your use of birth control. This information will help to improve health services for women with CF. So please take whatever time you need to answer them as accurately and completely as possible. Birth control includes methods that some people use to prevent pregnancy, to reduce cramping or heavy periods, to treat acne, or to prevent sexually transmitted disease. Since we've already discussed surgeries such as tubal ligation, we won't be asking about those methods again.

We understand you may have used multiple forms of BC at the same time, so dates may overlap. For example, some people use condoms at the same time as hormonal birth control.

Some things to consider if you are struggling to remember when you started or stopped a particular method of birth control: Were you dating someone or married? In school? Starting a new job? Living in a particular house or apartment? What reason did you have to start/stop taking it?

The next questions are asked for up to 5 episodes of use for each birth control method. Survey includes description, brand names (if applicable), and image(s) for each of the following methods:

1. Birth control pills containing both estrogen and progesterone

   Some brands include Alesse, Apri, Aranelle, Aviane, Azurette, Beyaz, Caziant, Desogen, Enpresse, Estrostep Fe, Gianvi, Kariva, Lessina, Levlite, Levora, Loestrin, Lybrel, Mircette, Mononessa, Natazia, Nordette, Ocella, Low-Ogestrel, Lo Ovral, Ortho-Novum, Ortho Tri-Cyclen, Previfem, Reclipsen, Safyral, TriNessa, Velivet, Yasmin, and Yaz.

Most birth control pills involve taking active pills for 21-24 days, and filler pills for 4-7 days, so you get your period once a month. Some women choose to take active pills continuously and skip a period all-together.

Some pills involve taking active pills for 3 months, and filler pills for 1 week, so you get your period once every 3 months. Some women choose to take active pills continuously and skip a period all-together.

These brands include Seasonale, Seasonique, and Jolessa.

2. Contraceptive patch

   This skin patch is worn on the lower abdomen, buttocks, or upper body (but not on the breasts). This method is prescribed by a doctor. It releases hormones progesterone and estrogen into the bloodstream. You put on a new patch once a week for three weeks. During the fourth week, you do not wear a patch, so you can have a menstrual period.

   Brands include Xulane and Ortho Evra

3. Vaginal ring

   The ring releases the hormones progesterone and estrogen. You place the ring inside your vagina. You wear the ring for three weeks, take it out for the week you have your period, and then put in a new ring.

   Brands include NuvaRing and Annovera.

4. Birth control pills containing progesterone only (“mini-pill”)

   Brands include Camila, Errin, Heather, Jolivette, Nor-QD, Ortho Micronor, Ovrette, and Tulana.

5. Copper IUD (non-hormonal IUD)

   This IUD is a small device that is shaped in the form of a "T." Your doctor places it inside the uterus to prevent pregnancy. It can stay in your uterus for up to 10 years.

   Brands include ParaGard.

6. Hormonal IUD

   This IUD is a small T-shaped device. It is placed inside the uterus by a doctor. It releases a small amount of progesterone each day to keep you from getting pregnant. The IUD stays in your uterus for up to 3 to 6 years, depending on the brand.

   Brands include Kyleena, Liletta, Mirena, and Skyla.

7. Arm implant

   The implant is a single, thin rod that is inserted under the skin of your upper arm. The rod contains a progesterone that is released into the body over 3 years.

   Brands include Implanon and Nexplanon.

8. Depo-Provera shot

   The doctor gives a shot of the hormone progesterone in the buttocks or arm every 3 months.

9. Condoms

Sometimes called “male” or “female” condoms. Male condom brands include Skyn, Durex, Trojan, Sustain, Okamoto, Pasante, GLYDE, and Kimono, and female condom brands include FC2.

10. Diaphragm or cervical cap

    These barrier methods are placed inside the vagina to cover the cervix to block sperm. The diaphragm is shaped like a shallow cup. The cervical cap is a thimble-shaped cup. Before sexual intercourse, you insert them with spermicide to block or kill sperm. Diaphragms and cervical caps come in different sizes, so a doctor's visit for a proper fitting is recommended.

    Brands include Caya, Janssen Ortho-All Flex, and FemCap.

11. Spermicide

    Includes gel, foam, suppository, or Today sponge. Spermicides work by killing sperm and come in several forms-foam, gel, cream, film, suppository, or tablet. They are placed in the vagina no more than one hour before intercourse. You leave them in place at least six to eight hours after intercourse. You can use a spermicide in addition to a male condom, diaphragm, or cervical cap.

    The contraceptive sponge contains spermicide and is placed in the vagina where it fits over the cervix. The sponge works for up to 24 hours, and must be left in the vagina for at least 6 hours after the last act of intercourse, at which time it is removed and discarded.

    Brands include Contragel, Delfen, Encare, Gynol II, VCF, and Today.

12. Withdrawal, or “pulling out”

    The withdrawal method of contraception is the practice of withdrawing the penis from the vagina and away from a woman's external genitals before ejaculation to prevent pregnancy.

13. Fertility awareness method (period-tracker app, rhythm method, natural family planning, cycle beads)

    This method can help you plan to get pregnant or avoid getting pregnant. Your fertility pattern is the number of days in the month when you are fertile (able to get pregnant), days when you are infertile, and days when fertility is unlikely, but possible. If you have a regular menstrual cycle, you have about nine or more fertile days each month. If you do not want to get pregnant, you do not have sex on the days you are fertile, or you use a barrier method of birth control on those days.

14. Breastfeeding for the first 6 months of an infant's life postpartum as a form of birth control

    For women who have recently had a baby and are breastfeeding, this can be used as birth control when three conditions are met: 1) not getting a period, 2) fully or nearly fully breastfeeding, and 3) less than 6 months after delivering a baby. This is a temporary method of birth control.

15. Primary sexual partner who is/was unable to physically impregnate you (e.g., female sex partner, had vasectomy, sterile)

16. Abstinence (refraining from sexual intercourse) as a form of birth control

In your lifetime, have you used [birth control method]?	Select one:<br>*1. Yes*<br>*2. No* [continue to next method]
If (1) selected<br>*Do you know the month and year when you started using [birth control method]?*	Select one:<br>*1. Yes* [enter month and year]<br>*2. No*
If (2) selected<br>*Do you know the year when you started using [birth control method]?*	Select one:<br>*1. Yes* [enter year]<br>*2. No*
If (2) selected<br>*Do you know how old you were when you started using [birth control method]?*	Select one:<br>*1. Yes* [enter age]<br>*2. No*
Did you ever stop using [birth control method]?	Select one:<br>*1. Yes*<br>*2. No* [continue to next method]
If (1) selected<br>*Do you know the month and year when you stopped using [birth control method]?*	Select one:<br>*1. Yes* [enter month and year]<br>*2. No*
If (2) selected<br>*Do you know the year when you stopped using [birth control method]?*	Select one:<br>*1. Yes* [enter year]<br>*2. No*
If (2) selected<br>*Do you know how old you were when you stopped using [birth control method]?*	Select one:<br>*1. Yes* [enter age]<br>*2. No*
Did you use them again at a later time [birth control method]?	Select one:<br>*1. Yes* [repeat questions for next episode of use, up to 5 episodes]<br>*2. No* [continue to next method]

Emergency Contraception

In your lifetime, have you used Emergency contraception (morning after pills)?<br>[includes description, brand names, and images]	Select one:<br>*1. Yes*<br>*2. No*
If (1) selected<br>*Approximately how many times since 2008 have you used emergency contraception?*	

Respondent Certainty

On a scale of 1-4, 1 being very uncertain and 4 being completely certain, how confident are you of the accuracy of your responses about your birth control history?	Select one:<br>*1. Very uncertain*<br>*2. Somewhat uncertain*<br>*3. Somewhat certain*<br>*4. Very certain*

Pregnancy History

The next section is about your experience with childbearing and pregnancy. This information will help to improve health services for women with CF. We understand this may be a sensitive topic for some people, so please take whatever time you need to answer them as accurately and completely as possible. We will be asking you about any pregnancies you have had - whether they resulted in:

- Live births;
- Miscarriage;
- Stillbirth;
- Abortion, or;
- Ectopic or tubal pregnancy.

Have you ever been pregnant? Remember, this should include all pregnancies: live births (babies born alive), miscarriage, abortion, stillbirth, or ectopic or tubal pregnancies.	Select one:<br>*1. Yes*<br>*2. No* [end of survey]<br>*3. Prefer not to answer* [end of survey]
If (1) selected<br>*What was the result of your most recent pregnancy?*	Select one:<br>*1. Live birth*<br>*2. Miscarriage (20 weeks or fewer/ less than 6 months)*<br>*3. Stillbirth (21 weeks or more/ 6 months or more)*<br>*4. Ectopic or tubal pregnancy (pregnancy occurs outside the uterus)*<br>*5. Abortion (for any reason, including because of health risks)*<br>*6. Prefer not to answer*
When did this pregnancy end?	Select one:<br>*1. I know the month and year*<br>*2. I know the year*<br>*3. I know how old I was*<br>*4. I don't know*
If (1) selected<br>*What month did this pregnancy end?*	
If (1) or (2) selected<br>*What year did this pregnancy end?*	

If (3) selected<br>*How old were you when this pregnancy ended?*	
Was this pregnancy planned or unplanned?	Select one:<br>*1. Planned*<br>*2. Unplanned*
Did you use assisted reproductive technology, like IVF or artificial insemination, for this pregnancy?	Select one:<br>*1. Yes*<br>*2. No*
During this pregnancy, did you visit your CF center?	Select one:<br>*1. Yes*<br>*2. No*
If (1) selected<br>*How frequently did you visit your CF center during this pregnancy?*	Select one:<br>*1. Once*<br>*2. Quarterly (every few months)*<br>*3. Monthly*
During this pregnancy, were you treated by any of the following healthcare professionals?	Select all that apply:<br>*1. OB/GYN*<br>*2. High risk OB/GYN (aka Maternal Fetal Medicine Specialist)*<br>*3. Midwife*<br>*4. Family Practice doctor*<br>*5. Other*<br>*6. Unsure/Don't know*<br>*7. None of the above*
If (5) selected<br>*Please specify other healthcare professional type*	
Was that (were any of the) healthcare professional(s) associated with the same hospital as your CF center during this pregnancy?	Select one:<br>*1. Yes*<br>*2. No*<br>*3. Unsure/Don't know*
**Previous Pregnancy**<br>*Was there another pregnancy prior to this one? Remember, this should include all pregnancies: live births (babies born alive), miscarriage, abortion, stillbirth, or ectopic or tubal pregnancies.*	Select one:<br>*1. Yes* [repeat questions for previous pregnancy, up to 5 pregnancies]<br>*2. No* [end of survey]
